# Factors associated with self-reported first sexual intercourse in Scottish adolescents

**DOI:** 10.1186/1756-0500-2-42

**Published:** 2009-03-19

**Authors:** Suzanne C Penfold, Edwin R van Teijlingen, Janet S Tucker

**Affiliations:** 1 Department of Palliative Care, Policy and Rehabilitation, King's College London, Weston Education Centre, Cutcombe Road, London, UK; 2 Infectious Disease and Vector Biology Unit, Department of Infectious and Tropical Diseases, London School of Hygiene and Tropical Medicine, Keppel Street, London, UK; 3 The Dugald Baird Centre for Research on Women's Health, Institute of Applied Health Sciences, University of Aberdeen, Foresterhill, Aberdeen, UK; 4 School of Health & Social Care, Bournemouth University, UK

## Abstract

**Background:**

There is continuing concern about high pregnancy rates and increasing numbers of sexually transmitted infections being detected in Scottish adolescents. Consistent evidence about factors associated with risky sexual behaviours, including early first sexual intercourse, may help to identify adolescents at risk and help improve interventions. This study aimed to provide detailed analysis of the evidence of the associations between individual factors and early sexual intercourse using cross-sectional questionnaire data from 4,379 Scottish adolescents who participated in a sexual health intervention evaluation.

**Findings:**

Multivariate secondary analysis showed that aspects of family and school life such as decreasing parental monitoring (OR 1.45, 95% CI 1.24–1.70) and decreasing enjoyment of school (OR 2.55, 95% CI 2.15–3.03) were associated with reporting previous sexual intercourse. Furthermore, females were more likely to report previous sexual intercourse than males (OR 1.48, 95% CI 1.14–1.91). Several factors commonly used to inform sexual health intervention design, such as socioeconomic status, self-esteem and religion, were not independently associated.

**Conclusion:**

These results contribute to the evidence base for the association of several factors with early initiation of sexual activity. The findings suggest that interventions aiming to delay first intercourse may need to consider targeting aspects of individuals' connection to their school and family. Furthermore, the results do not support the need to consider socio-economic background, religion or self-esteem of the individuals in intervention design.

## Background

There is continuing concern about adolescent involvement in risky sexual behaviours in the UK [1], particularly in Scotland because of increasing rates of sexually transmitted infection diagnoses and high pregnancy rates (56.7 pregnancies (births and abortions) per thousand women age 15–19 in 2005) compared to other European countries [2,3].

A younger age of first sexual intercourse is associated with greater sexual risk-taking, such as poor contraceptive use [4]. Therefore delaying first sexual intercourse may reduce sexual risk-taking and adverse sexual health outcomes. Consistent evidence about factors associated with risky sexual behaviours, including early first sexual intercourse, may help to identify adolescents at risk and help improve interventions. Although evidence demonstrates many factors associated with adolescent sexual risk-taking, the direction and strength of these associations vary. This study aimed to provide further evidence of factors associated with early first sexual intercourse using cross-sectional data from 4,379 Scottish adolescents who participated in a sexual health intervention evaluation.

## Methods

A cross-sectional school-based survey collected data from 16 secondary schools in the Lothian and Grampian regions of Scotland in 2003 which formed the follow-up part of the external evaluation of Healthy Respect Sexual Health and Relationships Education (SHARE), a national health demonstration project [5]. Ten Lothian schools that had agreed to implement the new Healthy Respect SHARE programme from January 2002 were selected for the evaluation. A comparison sample of Grampian (non-SHARE) schools were selected to participate, seeking to match with individual Lothian SHARE schools using routine data about school size, rurality, and proportion of pupils with free school meals. Six schools in Grampian agreed to participate in the follow-up evaluation. All participating schools in both Lothian and Grampian were nondenominational state secondary schools, representing city and town settings (including smaller towns with rural catchments), and with varying levels of deprivation and school size [5].

Eligible participants were all pupils in academic years S3 and S4 (Scottish secondary school years 3 and 4), who are usually aged between 14 and 15 years, at the 16 participating schools, and whose parents had been given the opportunity to opt their child out of the evaluation. Pupils were informed about the study three weeks before the survey and their written consent was sought before administering the questionnaire.

Data were collected using an adapted SHARE questionnaire . Trained research assistants administered anonymous questionnaires at school under exam conditions. There were 4,381 questionnaires completed (response rate 84.4%); two spoilt questionnaires were excluded. Non-response was mainly due to absenteeism (12.2%), rather than non-consent from parents/pupils (3.5%).

Secondary analysis was undertaken using the statistical analysis package Stata v10 [6]. Reported sexual intercourse was the primary binary outcome variable, with the assumption that first sexual intercourse in this age group was 'early' (the legal age of consent is 16 years in Scotland). Explanatory variable classification and selection from the questionnaires was based on published evidence of association with risky sexual behaviours in adolescence, including early first sexual intercourse [7-15]. Of the factors identified, those which had been measured in the questionnaire are shown in Table 1. The original total sample size requirement of 4000 pupils for the evaluation was estimated to be able to detect a 5% difference in reported rates of previous sexual intercourse in this age range, at 20% power and probability of 0.05. Recommendations to guide sample size estimates for multiple regression modelling suggest that the number of variables within each model should be less than the square root of the sample size, or that they should be less than 10% of the sample size [16]. As we planned to fit sixteen variables into the predictive model, the sample size of over 4000 was adequate for this purpose.

**Table 1:** Characteristics of the study population

Variable	Population characteristics
*Categorical variables*	
Gender	
Male	52%
Female	48%
Ethnicity	
White	97.3%
Non-white	2.7%
Family type	
Natural parents	64.7%
Natural parent + step parent	12.6%
Single parent	21.5%
Grand/foster parents	1.2%
Number of siblings	
Single child	7.4%
Not single child	92.6%
Religion	
None	62.6%
Christian	36.6%
Other	1.3%
Religiosity	
Religious	9.6%
Not religious	90.4%
Average weekly spending money (from pocket money or work)*	
<£10	22.6%
£10–20	60.2%
>£20	17.2%
Type of housing	
Owner occupied	7.9%
Rented/council	27.1%
Other/don't know	15.0%
Family employment	
Wage earner in family	87.8%
Unemployed	4.1%
Other non-wage earner	4.3%
Occupation not known	3.8%
Highest family educational attainment	
College/university	47.0%
Highers	6.9%
Standard grades	10.9%
Left school age 16	14.1%
Don't know	21.1%
Feel able to talk with parents about private matters	
Both parents	17.9%
Either parent	34.3%
Neither parent	47.9%
Aspirations to continue education after age 16	
Very likely/likely	70.5
Unsure/unlikely/very unlikely	29.5%
*Continuous variables*	
Age (range 13.1–16.5 years)	Mean 14 years 8 months (sd 0.6 years)
Self-esteem (range 1–4 where 1 is higher self esteem)	Median score 2.0 (interquartile range (IQR) 1.8–2.5)
Parental monitoring (range 1–4 where 1 is higher parental monitoring)	Median score 2.3 (IQR 1.5–2.8)
Enjoyment of school (range 1–4 where 1 is higher enjoyment of school)	Median score 2.0 (IQR 1.7–2.3)

Most explanatory variables were categorical. The last three variables listed in Table 1 were scores derived from combining responses to a number of related categorical questions. All scores generated were normally distributed, had inverse scales, and they had Cronbach's alpha co-efficients ranging from 0.58 to 0.78, indicating adequate reliability.

Bivariate logistic regression analyses of the 16 variables were computed to identify those that were significantly associated with the likelihood of reported sexual intercourse (p < 0.05). Robust standard errors were used throughout owing to the clustered nature of the data.

The same variables were entered into multiple logistic regression models using the forward likelihood ratio (LR) selection procedure, excluding missing cases. There was little difference between the bivariate odds ratios for the whole population and those for the complete cases included in the multiple models in the values, indicating minimal response bias in the variables included in the models. This allowed comparisons between the bivariate and multivariate odds ratios to be made.

## Results

Of the 4,379 respondents, 52% were male and 48% female. The age range was 13.1 to 16.5 years, with a mean of 14 years 8 months (standard deviation (SD) = 0.6 years). Nearly 22% (n = 909) of respondents reported previous sexual intercourse. The majority was White, lived with both natural parents and at least one sibling in an owner-occupied house, had at least one parent in paid employment and one parent with college/university education, followed no religion and were not religious, and aspired to continue education after age 16. Nearly half (47.9%) felt unable to talk to either parent about private matters. Overall, respondents' scores demonstrated positive self-esteem, enjoyment of school and parental monitoring. The population characteristics are presented in Table 1. Valid responses were received from at least 96% of participants for all variables, with the exception of spending money where valid responses were received from 91.7% of participants.

Bivariate logistic regression showed respondents reporting previous sexual intercourse were significantly more likely to: be older; be following no religion or have no religious beliefs; be not living with both biological parents; have more spending money each week; be living in council or rented housing; have unemployed parents or parents who left school at 16 (these latter three characteristics indicated lower socioeconomic status (SES)); be unable to talk to either parent about private matters; not aspire to continue education after age 16; and have significantly lower levels of self-esteem, parental monitoring and enjoyment of school. There was no significant difference in the likelihood of reporting sexual intercourse between genders, between Whites and non-Whites, or between lone children and those with siblings (Table 2).

**Table 2:** Factors significantly associated with reported sexual intercourse in bivariate logistic regression.

Variable	Odds Ratio (95% CI)
Age	2.15 (1.68, 2.75)***
Gender	
Male	1.00
Female	1.10 (0.91, 1.34)
Ethnicity	
White	1.00
Non-White	1.026
Family type	
Natural parents	1.00
Step parent plus natural	2.15 (1.66, 2.77)***
Single parents	1.56 (1.28, 1.91)***
Grand/foster parents	5.59 (2.93, 10.67)***
Number of siblings	
Single child	1.00
Not single child	0.91 (0.67, 1.21)
Religion	
None	1.00
Christian	0.76 (0.62, 0.94)*
Other	1.73 (0.89, 3.34)
Religiosity	
Religious/very religious	1.00
Unsure/Not religious/not at all religious	1.31(1.01, 1.69)*
Type of housing	
Owner occupied	1.00
Council/rented	1.54 (1.29, 1.84)***
Care/other/don't know	1.11 (0.80, 1.53)
Average weekly spending money	
<£10	1.00
£10–£20	3.16 (2.20, 4.54)***
>£20	5.49 (3.83, 7.89)***
Family employment	
Wage earner in family	1.00
Unemployed	1.72 (1.21, 2.43)**
Other non-wage earner	1.47 (0.90, 2.39)
Occupation not known	0.93 (0.66, 1.30)
Highest family education	
College/university	1.00
Highers/A-levels	1.24 (0.95, 1.64)
Standard grades/O-levels	1.22 (1.00, 1.48)
Left school <16	1.77 (1.44, 2.16)***
Don't know	1.05 (0.87, 1.29)
Decreasing self-esteem	1.24 (1.01, 1.53)*
Decreasing parental monitoring	2.12 (1.87, 2.40)***
Feel able to talk about private matters with:	
Both parents	1.00
Either parent	1.26 (0.94, 1.71)
Neither parent	1.95 (1.49, 2.55)***
Decreasing enjoyment of school	3.32 (2.79, 3.95)***
Aspirations to continue education after age 16	
Very likely/likely	1.00
Unsure/unlikely/very unlikely	1.78 (1.53, 2.08)***

* p < 0.05.

** p < 0.01.

*** p < 0.001.

After accounting for other influences, multivariate logistic regression analysis (Table 3) showed that respondents reporting previous sexual intercourse were significantly more likely to be older (OR 2.16 (95% CI1.73, 2.70)), not live with both biological parents (natural parents OR 1.00, step family OR 2.02 (95% CI 1.56, 2.64), single parent OR 1.33 (95% CI 0.98, 1.82), grand/foster parents OR 4.27 (95% CI 1.52, 11.98)), have received higher levels of spending money (<£10 OR 1.00, £10–£20 OR 2.85 (95% CI 2.19, 3.72) >£20 OR 3.65 (95% CI 2.65, 5.01)), be female (OR 1.48 (95% CI 1.14, 1.91)), and report lower levels of parental monitoring (OR 1.45 (95% CI 1.24, 1.70)) and enjoyment of school (OR 2.55 (95% CI 2.15, 3.03)). These variables explained about 15% of the variation in the likelihood of reported sexual intercourse.

**Table 3:** Multivariate logistic regression model of factors associated with reported sexual intercourse

Variable	Odds Ratios (95% CI)
Age	2.16 (1.73, 2.70)***
Family type	
Natural parents	1.00
Step family	2.02 (1.56, 2.64)***
Single parent	1.33 (0.98, 1.82)
Grand/foster parents	4.27 (1.52, 11.98)**
Average weekly spending money	
<£10	1.00
£10–£20	2.85 (2.19, 3.72)***
>£20	3.65 (2.65, 5.01)***
Gender	
Male	1.00
Female	1.48 (1.14, 1.91)**
Decreasing parental monitoring	1.45 (1.24, 1.70)***
Decreasing enjoyment of school	2.55 (2.15, 3.03)***

Number cases 3497/4379.

* p < 0.05.

** p < 0.01.

*** p < 0.001.

## Discussion

Nearly 22% of respondents reported previous sexual intercourse at a young age. Our study shows that adolescents who did not live with both biological parents, were female, received more spending money and had 'weaker' family relationships and school engagement had a greater likelihood of reporting previous sexual intercourse.

Socio-economic deprivation, aspects of religion and self-esteem were not associated with previous sexual intercourse whilst controlling for other factors.

These results contribute to the evidence base for the involvement of these factors in sexual behaviours. The importance of family and school in risky sexual behaviours [7,8,17] and the non-significant association between self-esteem and sexual intercourse [18] have been well documented. Although many studies have shown associations between risky behaviours and SES or religious domains (e.g. Henderson et al. 2002 [9]) in multivariate analysis, several others have not [19,20].

Our study had several strengths and weaknesses. First, the evaluation study from which the data were taken had a high response rate from a large population. Secondly, reported rates of sexual intercourse by pupils in our study were comparable to ones previously reported [8]. Thirdly, the variables selected for analysis all had previous evidence of association with risky sexual behaviour. Fourthly, as the age group of the study population was narrow the findings are relevant to the age group in question and may inform better intervention targeting, although the associations found are likely to be less generalisable to other age groups. Fifthly, the lack of religious affiliation and religiosity in notably non-denominational schools may mean that this factor has not been tested fully in this study. These results may be of relevance for the majority of Scottish schools that are non-denominational but we cannot exclude the possibility that the findings may be different in different countries or different school-type settings. Sixthly, the scores generated were reliable and some scores had been used in other analyses using similar items [9]. Seventhly, participants were asked to report their own behaviour, thus over- or underreporting of previous sexual activity may have occurred. Eighthly, the cross-sectional nature of the study means that only associations between factors and reported behaviour were demonstrated, not causation. However, the findings may still help to inform the targeting of interventions. Finally, the model produced explained only 15% of the variation in reported sexual intercourse in this population.

In conclusion, these results contribute to continuing debates around the important and complex association of several factors with early initiation of sexual activity. The findings suggest that interventions aiming to delay first intercourse may need to consider targeting aspects of individuals' connection to their school and family as well as gender. Furthermore, the results do not support the need to consider socio-economic background, religion or self-esteem of the individuals in intervention design in this age group of adolescents and in these types of non-denominational schools in Scotland.

## Competing interests

The authors declare that they have no competing interests.

## Authors' contributions

SCP carried out data collection, conducted analysis and drafted the manuscript. JST conceived the evaluation from which the data were obtained for secondary analysis, supervised SCP and contributed to the manuscript. EVT participated in the design of the evaluation, supervised SCP and contributed to the manuscript. All authors have given approval for this manuscript to be submitted.
